# New Diagnostic Tool for Ion Channel Activity Hidden Behind the Dwell-Time Correlations

**DOI:** 10.1021/acs.jpcb.2c02272

**Published:** 2022-06-02

**Authors:** Przemysław Borys, Paulina Trybek, Beata Dworakowska, Piotr Bednarczyk, Agata Wawrzkiewicz-Jałowiecka

**Affiliations:** † Department of Physical Chemistry and Technology of Polymers, Silesian University of Technology, 44-100 Gliwice, Poland; ‡ Faculty of Science and Technology, University of Silesia in Katowice, 41-500 Chorzów, Poland; ¶ Institute of Biology, Department of Physics and Biophysics, Warsaw University of Life Sciences - SGGW, 02-787 Warszawa, Poland

## Abstract

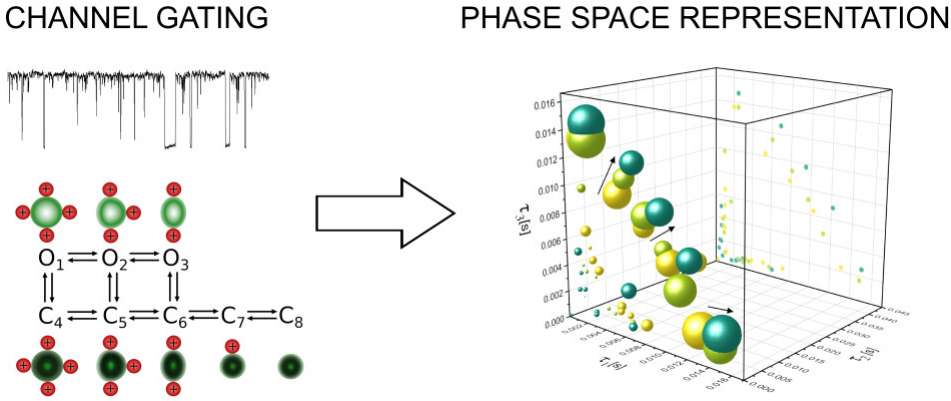

The patch-clamp technique is a powerful tool that allows for a long observation of transport protein activity in real time. Experimental traces of single-channel currents can be considered as a record of the channel’s conformational switching related to its activation and gating. In this work, we present a mathematically simple method of patch-clamp data analysis that assesses the connectivity and occupancy of distinct conformational substates of the channel. The proposed approach appears to be a big step forward due to its possible applications in the determination of channel substates related to disease and in the analysis of drug–channel interactions on the level of repetitive sequences of channel conformations. This is especially important in cases when molecular dynamics docking is impossible and Markovian modeling requires ambiguous optimization tasks.

## Introduction

Single-molecule electrophysiological techniques such as the patch-clamp^1^ and the related signal analysis continue to be the most powerful experimental approaches to study ion channels. They are capable of capturing thousands of channel openings and closings at a temporal resolution on the order of tens of microseconds. Consequently, electrical activity of a small patch of a biological membrane can deliver unique biological information on conformational changes of individual transport proteins in real time.

### Patch-Clamp Signal Characteristics

**Figure 1:**
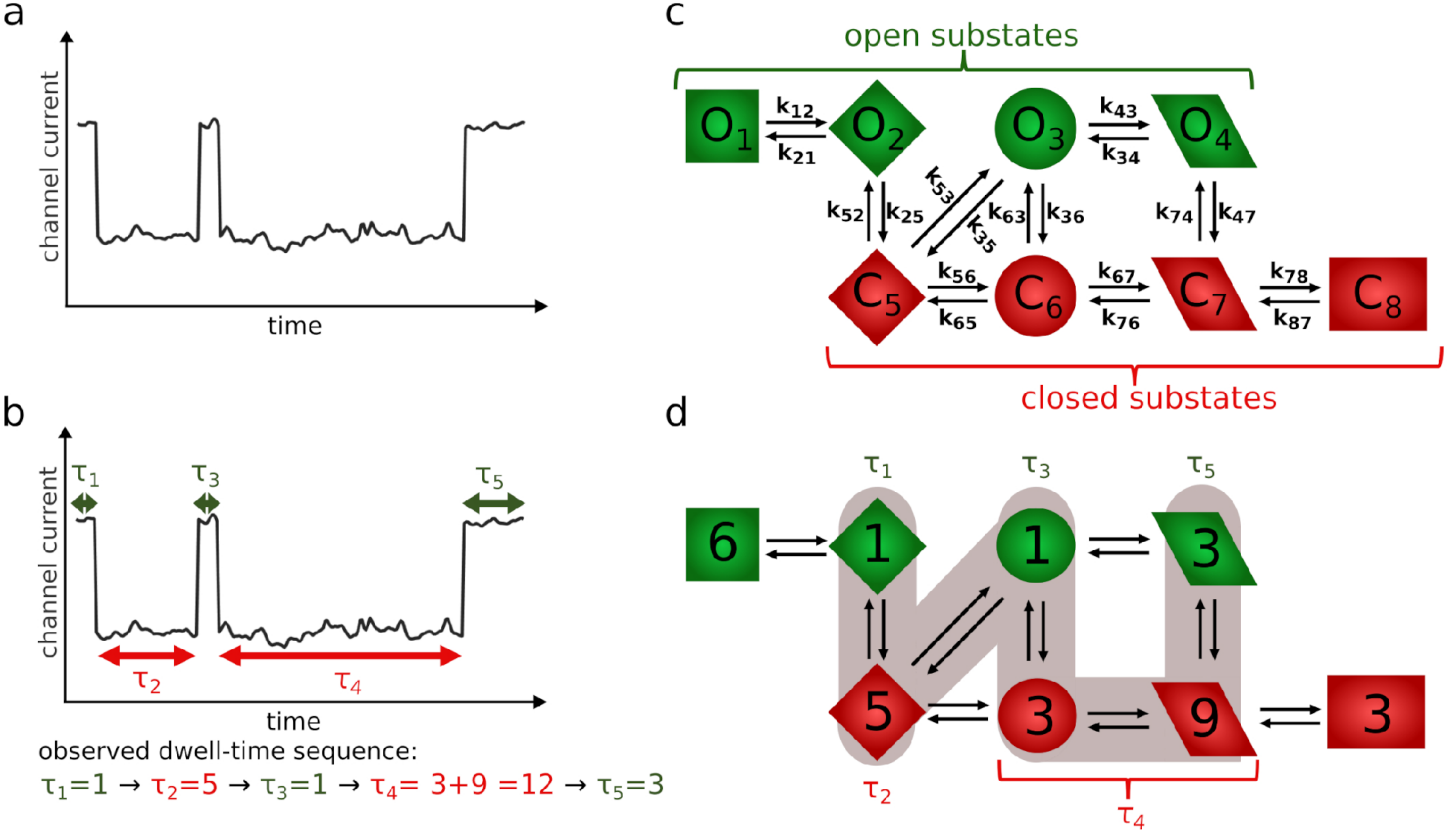
An arbitrarily chosen sample representation of the patch-clamp signal as a time series of the single-channel current (a). On the basis of the channel current value, the actual functional state can be identified. Here, we consider two types of channel states, i.e., open (green) and closed (red). Thus, for the experimental patch-clamp recording, a corresponding series of dwell-times (τ) of open and closed channel states can be found (b). Channel gating kinetics is typically represented by a diagram where stable channel conformations (substates) are connected according to their energetically and spatially available transitions with rate constants *k*_*ij*_, as presented for an exemplary case in (c). An example trajectory of dwell-times (gray trace in (d)) corresponding to the recorded single-channel patch-clamp signal (a) is presented within a kinetic diagram of a given channel. Numbers denote the expected (average) dwell-times for particular substates that were symbolically denoted as O_1_–C_8_ in (c). To highlight that the substates O_1_–C_8_ directly correspond to different stable conformations of a channel, we used different shapes and colors in their symbolic representation (c, d).

The empirical patch-clamp data are typically given in the form of a time series of single-channel currents,^1^ as shown in Figure 1a. On the basis of the original recording, one can straightforwardly construct the corresponding series of dwell-times in successive functionally discernible states (Figure 1b). In most cases, these are the conducting (open) and nonconducting (closed) states, as presented in Figure 1 using green and red colors, respectively. Under a fixed set of external conditions, there is some number of stable channel conformations (here also called substates) that can be classified as open/closed states (Figure 1c). In general, the switching of the channel between substates follows some particular, energetically favorable, conformation change pathway. This kind of behavior can be traditionally represented by an aggregated Markov process. The possible changes between the channel’s substates are reflected by the connectivity of substates within a kinetic scheme of a Markov type, as symbolized by arrows in Figure 1c,d. Each individual experimental dwell-time may represent the persistence of the channel in one stable channel conformation (a single substate), or it may correspond to a sequence of consecutive conformations (substates), which functionally correspond to the same state. The second case is depicted in Figure 1 by observation of the dwell-time (τ_4_ = 12), a composition of two closed substates lasting 3 and 9 time units. Thus, a sole dwell-time within the experimental record is not sufficient to unequivocally identify to which substate (or set of substates) it corresponds in the kinetic scheme.

### Repetitiveness of Dwell-Time Sequences

The central idea of the methodology presented here mostly focuses on correlations between the short dwell-time sequences of the subsequent channel states. The basic observations underlying our findings are that stable conformations of a channel must appear multiple times in the patch-clamp recording, and there is a limited number of the possible connections between the subsequent channel substates. If that is the case, it will likely result in repetitions of similar dwell-time sequences in the analyzed experimental data.

Generally, the dwell-time of a given state depends on the duration of the previous state, which indicates the presence of correlations between these open and closed dwell-times.^2−5^ Let us recall that the Markov process, which usually represents the kinetics of conformational switching of the ion channel (Figure 1c), is memoryless. This means that the probability of the next substate depends only on the current substate of a given system. Nevertheless, the existence of correlations between the channel’s states can be interpreted even in terms of the “classic” Markovian framework by the use of multistate models of complex connectivity and the presence of loops within the kinetic schemes.^3^ The novelty of our approach is established by the practical direct utilization of the repetitiveness and correlations between the dwell-times in the description of the ion channel activity. The repetitious sequences of a similar state can refer to the particular changes between the channel’s conformations in a unique way.

Namely, the set of routes allowing a given channel’s substate to enter and exit should enable the identification of a particular substate within experimental data with a high degree of certainty. For example, in Figure 1c,d, the C_6_ substate of dwell-time 3 can be followed by the C_7_ that dwells 9 units and vice versa. In these terms, one can observe the effective channel’s closing for 12 time units. How can the composition of the substates C_6_ and C_7_ be distinguished from C_7_ and C_6_? The first variant (C_6_, C_7_) can be reached from the sequence C_5_–O_3_– lasting 5–1–, which is not available for the second one (C_7_, C_6_), while the second of them (C_7_, C_6_) can be reached from the sequence C_5_–O_3_–O_4_– where the proceeding closed and open states effectively dwell 5–4–, which is not available for the first combination (C_6_, C_7_). To distinguish any substates, it is enough when only one of the entry/exit routes differs.

The above findings could guide the reconstruction of the Markovian graph describing the channel. Some incomplete ideas were already formulated in a similar context.^2,3,6−8^ Here, we show that the main strategy to study the gating kinetics does not have to be based on the reconstruction of the Markov diagram representing a given channel. Instead, we propose the replacement of such a model just by a phase diagram in the “dual space” of conformations, which represents the correlated sequences of the subsequent dwell-times. Such a diagram is informative yet easier to analyze than the raw patch-clamp data.

### A Running Example: BK and mitoBK Channels

**Figure 2:**
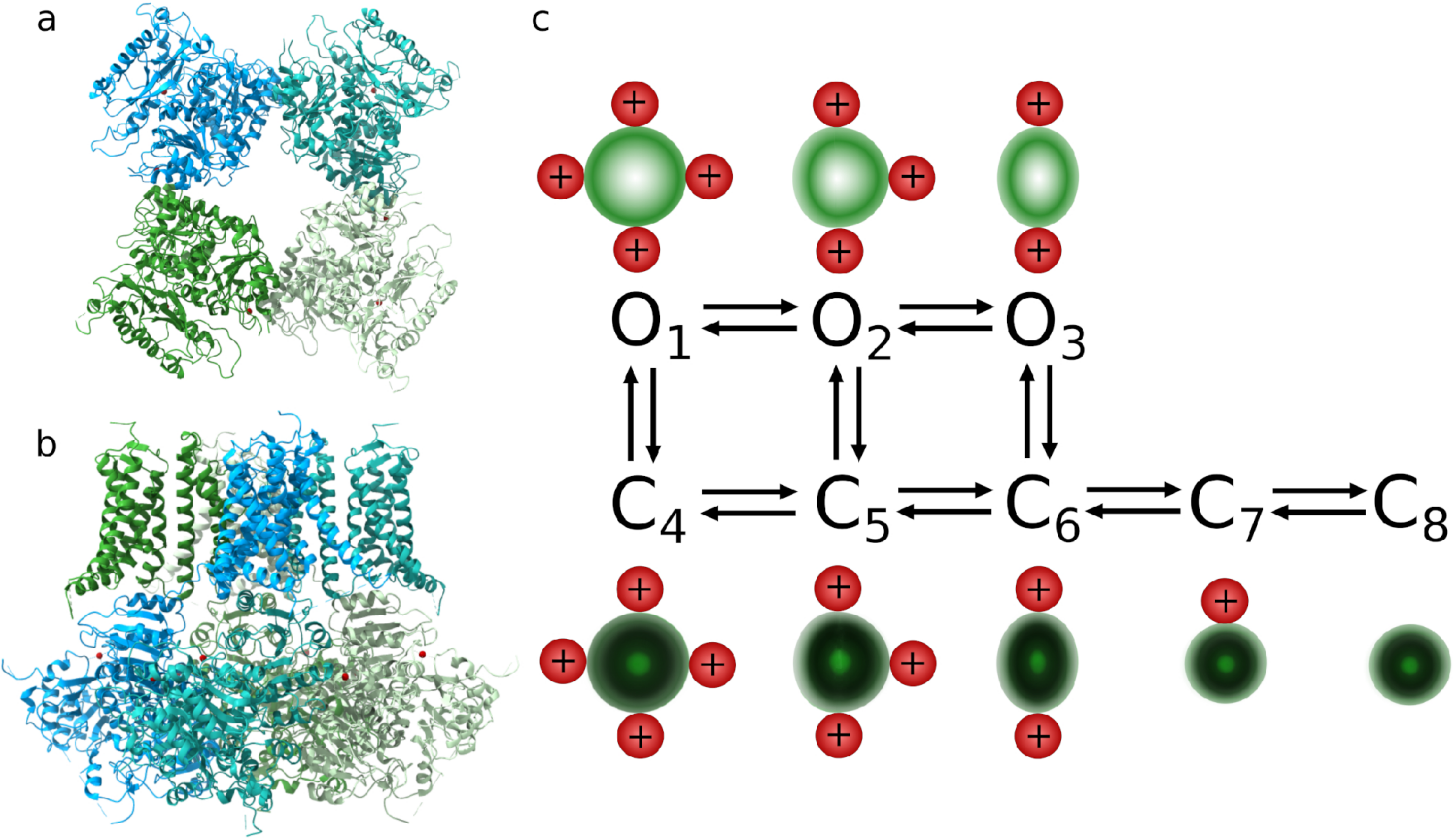
Ca^2+^-bound BK channel structure (PDB ID 6V22 sketched and analyzed in UCSF ChimeraX^15,16^). The frontal view on the gating ring from the cytoplasmic side (a). The side view on the α subunits of the BK channel (b). A possible schematic representation of the BK channel kinetics for fixed membrane potential (in its relatively low regime) and fixed Ca^2+^ concentration by 3 open (O_1_–O_3_) and 5 shut substates (C_4_–C_8_) (c). The gating ring (green) can account for the coordination of up to eight calcium ions: two Ca^2+^ ions coordinated by each α subunit (symbolically represented by red positively charged spheres). Activation of at least two calcium sensors effectively stretch the pore and, eventually, open it (white shading corresponds to the wide open entrance to the pore). The different amounts of bound calcium ions imply different conformational states of the channel (and different probabilities for retaining the pore open). The substate O_1_ corresponds to structures depicted in parts (a) and (b).

In this work, we address the introduced methodology to the activity of the large-conductance voltage- and Ca^2+^-activated K^+^ channels (BK) channels from the plasma membrane^9^ and their mitochondrial counterparts mitoBK.^10,11^ The BK channels are ubiquitously expressed potassium channels that are activated by membrane depolarization and cytosolic Ca^2+^ and exhibit a large single-channel conductance (ca. 300 pS).^9,12,13^ In the case of a fixed Ca^2+^ concentration and constant membrane potential (*U*_m_), the kinetic scheme of 3–4 open and 5–6 closed substates can represent BK channel gating (in Figure 2, we pictorially describe the differences between the available channel’s substates).^14^

The proposed methodology will be applied to the signals describing two BK channel variants: the plasma membrane variant and its mitochondrial counterpart (mitoBK), where the membrane potential is a parameter while other external conditions are fixed. In such a way, we establish whether it is possible to trace the voltage-imposed conformation changes in the dwell-time phase space, i.e., whether it is possible to see some regularities in the correlation cluster positions, and the voltage dependent cluster position changes.

## Experimental and Theoretical Methods

### Cell Culture and Mitoplast Preparation

In this work, we analyzed the activity of BK channels from the plasma membrane and the mitoBK channels from the inner mitochondrial membrane in the human endothelial cell line (EA.hy926). The cells were cultured in DMEM supplemented with 10% fetal bovine serum, 2 mM l-glutamine, 100 U/mL penicillin, 1% l-glutamine, 2% hypoxanthine hypoxanthine–aminopterin–thymidine, and 1% penicillin–streptomycin at 37 °C in a humidified atmosphere with 5% CO_2_. The cells were fed and reseeded every third or fourth day.

To prepare fresh mitochondria and subsequent mitoplasts, centrifugation and hypotonic swelling were carried out as described in ref (10). Mitoplasts were prepared by incubation in a hypotonic solution (5 mM HEPES, 100 μM CaCl_2_, pH 7.2) for approximately 1 min, and then, a hypertonic solution (750 mM KCl, 30 mM HEPES, and 100 μM CaCl_2_, pH 7.2) was subsequently added to restore the isotonicity of the medium. For each repeating patch-clamp experiment, a fresh mitoplast was used.

### Patch-Clamp Measurements

The patch-clamp experiments were carried out in inside-out single-channel mode. The currents were recorded using a pipet of borosilicate glass (Harvard, UK) with a resistance of 7–20 MΩ, which was pulled using a Narishige puller. In experiments on the BK channel from the plasma membrane, the symmetric isotonic solution, which contained 140 mM potassium gluconate, 10 mM KCl, 1 mM CaCl_2_, 0.9 mM EGTA, and 10 mM HEPES at pH 7.3–7.4, was used in the bath and glass pipet. The recordings of mitoBK channels’ activity were carried out in mitoplast-attached mode. In this case, the patch-clamp glass pipet was filled with an isotonic solution containing 150 mM KCl, 100 μM CaCl_2_, and 10 mM HEPES at pH 7.2.

The patch-clamp amplifier Axopatch 200B records the single-channel currents. The signals were low-pass filtered at a corner frequency of 1 kHz and sampled with Clampex software at a frequency of 10.00 kHz (i.e., at time intervals of 100.00 μs). The following values of pipet potentials were used in the experiments: −60, –40, –20, 20, 40, and 60 mV. The measurement error of the single-channel currents was Δ*I* = 1 × 10^–6^ pA, which was implied by the equipment. Each experimental time series comprised at least *N*_I_ = 1.1 × 10^5^ current values (the maximal length of the recording was *N*_I_ = 2.3 × 10^6^, and the majority of the current traces was over *N*_Icell._ = 1.0 × 10^6^ long for the BK channels; the majority of the mitoBK channel recordings had ca. *N*_Imitoch._ = 2.0 × 10^5^ data points). At each value of membrane potential, we recorded time series of single BK/mitoBK channel currents using 3–8 independent patches. The recordings were performed at room temperature (22 °C).

### Input Data Preparation

The input data for the introduced methodology are the dwell-time series of subsequent channel states together with the identification of the functional state. The input data can be constructed either from the experimental patch-clamp recordings of single-channel currents or from the simulation of a channel activity if only it allows one to obtain a time series describing a number of acts of switching between the channel’s conducting and nonconducting states. Practically, each dwell-time should be labeled as open (O) or closed (C) states of the channel. To obtain the dwell-time series from the experimental time series of single-channel currents, one has to find the current threshold value used to separate conducting (O) and nonconducting (C) states. In that aim, the procedure described in the work of Mercik et al.^17^ can be applied. Then, one should construct a list of all possible N-element C–O–C–... and O–C–O–... dwell-time subseries (also called sequences). The sequences starting from the open state will be compared only to those also starting from open state, and those starting from channel closure are compared to shut-starting ones.

The dwell-time distributions have exponential distributions that largely overlap. To distinguish between them, the lists of the C–O–C–... and O–C–O–... dwell-time sequences should be sorted according to the decreasing value of the product of the dwell-times forming each sequence. Thus, the search for the similar dwell-time subseries within the input data starts from the longest observed sequences.

### Finding Correlations

When searching for the correlated sequences, we use the value of cross-correlation *R*_*XY*_ between the dwell-time series *X*_*i*_ and *Y*_*i*_ as a similarity criterion. It is defined by the ratio of covariance to root-mean variance, i.e.,
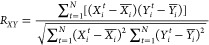
1

**Figure 3:**
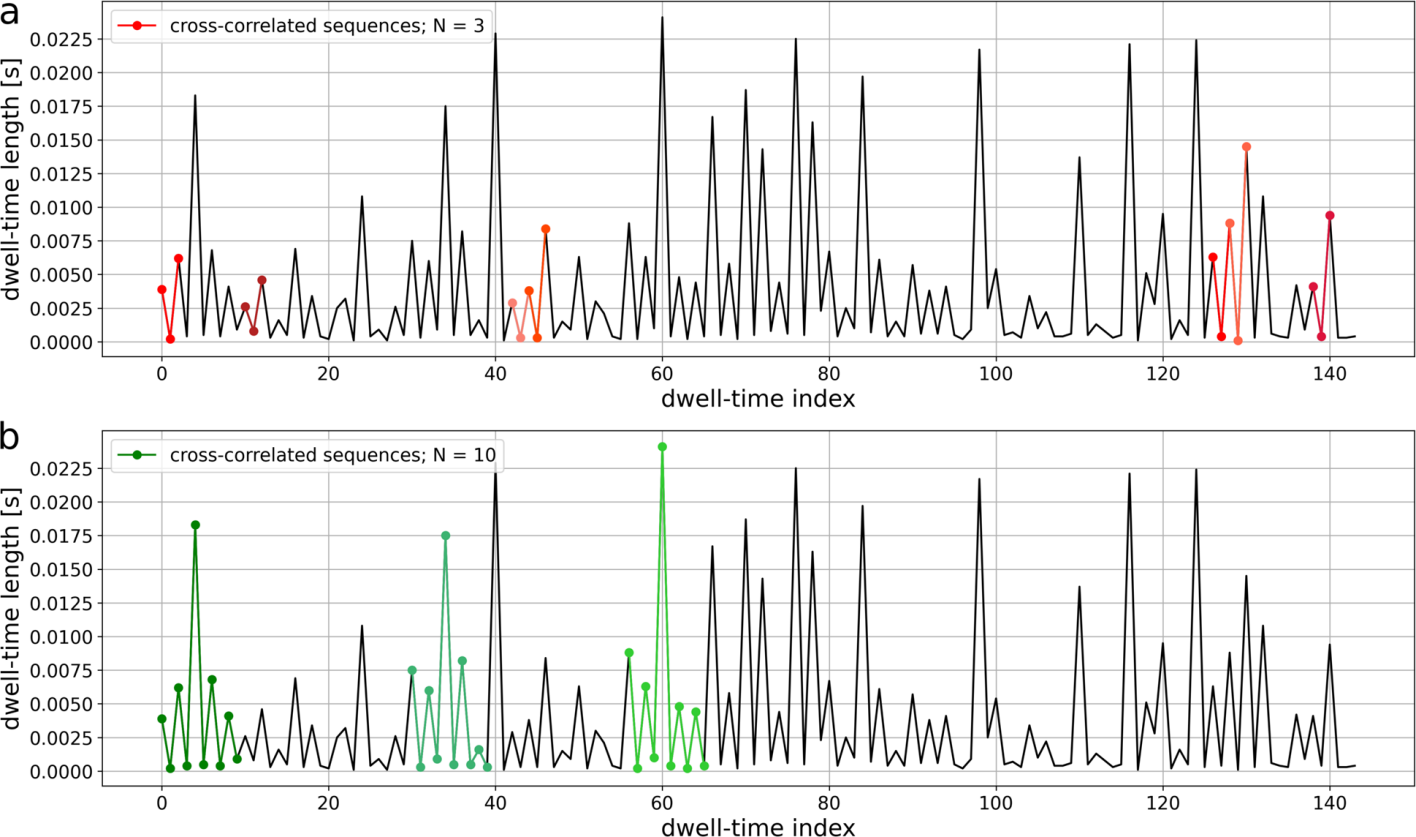
Examples of cross-correlated sequences of different lengths (*N*) presented on a sample dwell-time series. *N* = 3 (a); *N* = 10 (b). The data were obtained at a membrane potential of *U*_m_ = 20 mV for the BK channels from the plasma membrane of human endothelial EA.hy926 cells. To consider the analyzed dwell-time series to be sufficiently similar to each other, the cross-correlation threshold is set at *R*_0_ = 0.96. In these plots, it can bee seen that the correlated sequences are similar in shape and may be rescaled in amplitude, which reflects the broad, exponential distribution of dwell-times and postulated self-similarity of the data.^18,19^

The raw experimental data can often be considered as a noisy composition of basic events and unwanted perturbation. The basic events display a significant variability of dwell-times due to the exponential distribution of the waiting times. The cross-correlation criterion enables one to accept some inconsistencies of the recording after adjusting the cutoff threshold *R*_0_ and, due to the normalization by the standard deviations, to exploit the self-similarity of the patch-clamp data^18,19^ (see Figure 3).

The length of the investigated sequences *N* should be a compromise between the possibility to obtain a unique set of entry/exit sequences describing each channel’s substate and the limited length of experimental series that restricts the possibility to detect a statistically significant representation of a given sequence within the empirical data. As illustrated in Figure 3, with the increasing length of the sequence, the chance to encounter the cross-correlated sequences decreases (for *N* = 3, there are 7 exemplary correlated sequences within the analyzed excerpt of the dwell-time series (colored in shades of red (a)), whereas for *N* = 7, only 3 cross-correlated sequences were found (colored in shades of green (b))). The minimal value of *N* is 2 [the practical value is 3 (allows visualization)], while the maximal *N* is a number that allows for sufficient averaging of the sequences (we propose sequence sets having at least 10 representatives, covering more than 50% of the raw data). Using larger sequence lengths limits the possibility of visualization; nevertheless, the distances between the obtained clusters and changes in their position can still be measured by the multidimensional Pythagorean theorem and visualized effectively.

**Figure 4:**
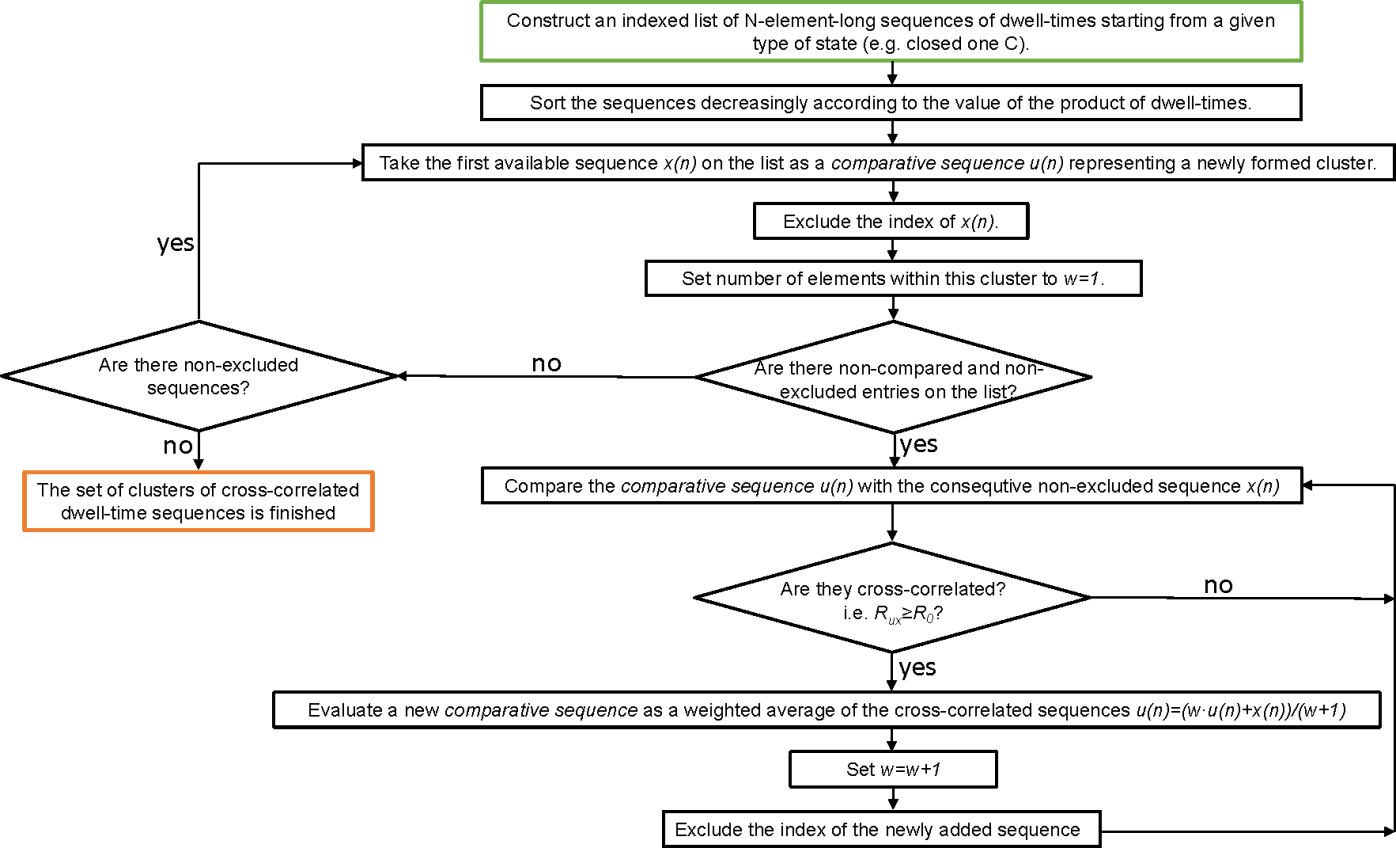
Flowchart explaining the consecutive steps of the proposed analysis of dwell-time sequences. The starting box is green-framed; the final one is orange-framed.

To sum up, the extraction of the sets of correlated dwell-time sequences should be carried out according to the procedure described by the flowchart in Figure 4 and consists of the following steps:

1. For a fixed value of *N* and the threshold value of cross-correlation, one should only consider the subseries, which start from the particular observable state of the channel, e.g., opening (i.e., the sequences of O–C–O–... type). The first sequence of this kind is given as the comparative series. We exclude its index (precisely, the index of its first element) in the input series.
2. The chosen comparative series is then compared with the next encountered (nonexcluded) sequence of this kind. According to the introduced cross-correlation criterion, if the cross-correlation value is higher than or equal to the introduced threshold *R*_0_, as a new comparative series, we take the weighted average of the considered subseries (the weight of the comparative sequence is the number of the sequences aggregated hitherto, initially 1; the weight of the added sequence is 1). Then, we exclude the index of the newly added sequence.
3. Step 2 should be executed (taking the next nonexcluded sequence) until the data are exhausted.
4. From the elements of the initial input series for which their indexes have not been excluded yet, one should take the first subseries and execute the operations from step 2. In this way, another set of similar sequences is generated for lower maximum tail dwell-times (since the higher ones are excluded).
5. Steps 2–4 should be repeated until the input data are exhausted.
6. All the aforementioned steps should be repeated for the sequences starting from another state (here, C–O–C–...).
7. Due to the adaptive changes in cluster templates in the search for correlations, after the initial data separation for each cluster, one should check whether all sequences included reach the *R*_0_ threshold to the final template sequence. If insufficiently correlated, they should be incorporated into another cluster for which they exhibit the cross-correlation ≥*R*_0_ or they should form a seed for another cluster of sequences. After the rejection/addition of a given sequence from a cluster, the corresponding template sequence should be appropriately updated.
8. The previous step should be repeated until reaching the final data separation of the clusters where no more corrections are needed.

Finally, our method allows one to obtain sets (clusters) of repeating correlated sequences of dwell-times of the channel states (of the O–C–O–... and C–O–C–... types) that can be plotted in the *N*-dimensional phase space and characterized by the cardinality (which reflects the frequency of occurrence), dispersion, the distances between the set centers, etc. The identification of the mostly occupied channel states and monitoring the changes in their location in phase space with the alteration of external conditions reflect the mechanism underlying the channel’s conformational dynamics.

### Search for the Optimal Correlation Threshold Value

The lower limit of *R*_0_ is restricted by a demand that the obtained sets show the statistical differences between each other. The upper limit for the *R*_0_ restricts the condition that the data within a cluster should be produced by a unique transition route. This can be tested by the use of exponential fitting: it is preferred that the distribution of sequences forming a cluster is monoexponential in all *N* dimensions.

To find the optimal value of the correlation threshold, we screened the *R*_0_ between 0.80 and 0.97 with step Δ*R*_0_ = 0.05 (and by Δ*R*_0_ = 0.01 for fine-tuning). For each set of correlated sequences at given *R*_0_ (only sets with over 50 samples considered), the single and double exponential fittings were performed for the probability density function (pdf) of its coordinates along all dimensions. The fit is accepted if the curve agreed with the experimental data within its uncertainty.

As a criterion for the choice of the optimal *R*_0_, we assumed the value for which the ratio of double exponential fits to the single exponential fits attains the minimum. In the case of limited amounts of data (e.g., below 6000 sequences), one can encounter problems with a relatively high proportion of the poorly occupied clusters especially at high *R*_0_. In such cases, it is recommended to consider only the results obtained at such values of *R*_0_ that allow 75% of the highly occupied sets (having at least 50 representatives) to be reached.

## Results

**Figure 5:**
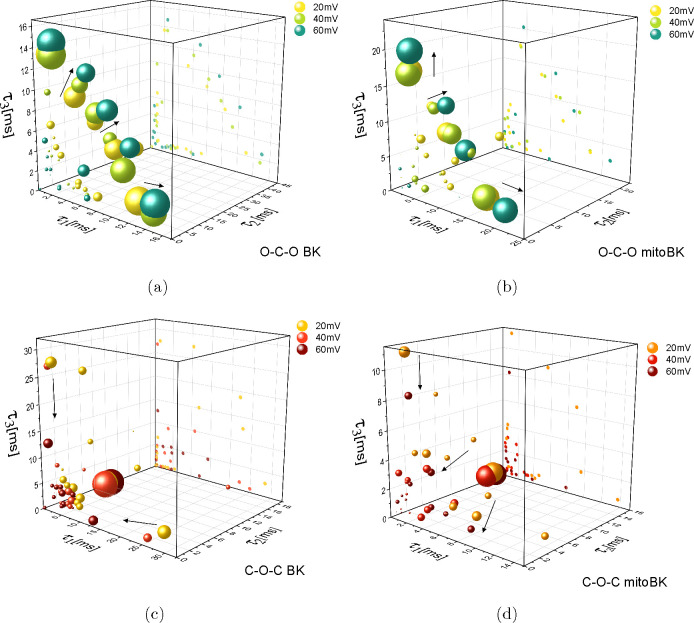
Phase space representation of the clusters of the O–C–O (a, b) and C–O–C (c, d) sequences describing channel gating at membrane depolarization for the plasma membrane BK channel variant (a, c) and its mitochondrial isoform mitoBK (b, d). The results are obtained at *R*_0_ = 0.96 (BK) and *R*_0_ = 0.93 (mitoBK). The coordinates of the centers of the spheres representing the O–C–O sequences are given by the average values of the dwell-times of cross-correlated sequences forming a given cluster. The volumes of the spheres are proportional to the logarithm of its normalized and rescaled cardinality. To simplify the recognition of the main directions of cluster translations with membrane potential, they are marked with black arrows and the centers of the clusters are orthographically projected on the τ_1_τ_3_ plane and presented by dots in the background.

**Figure 6:**
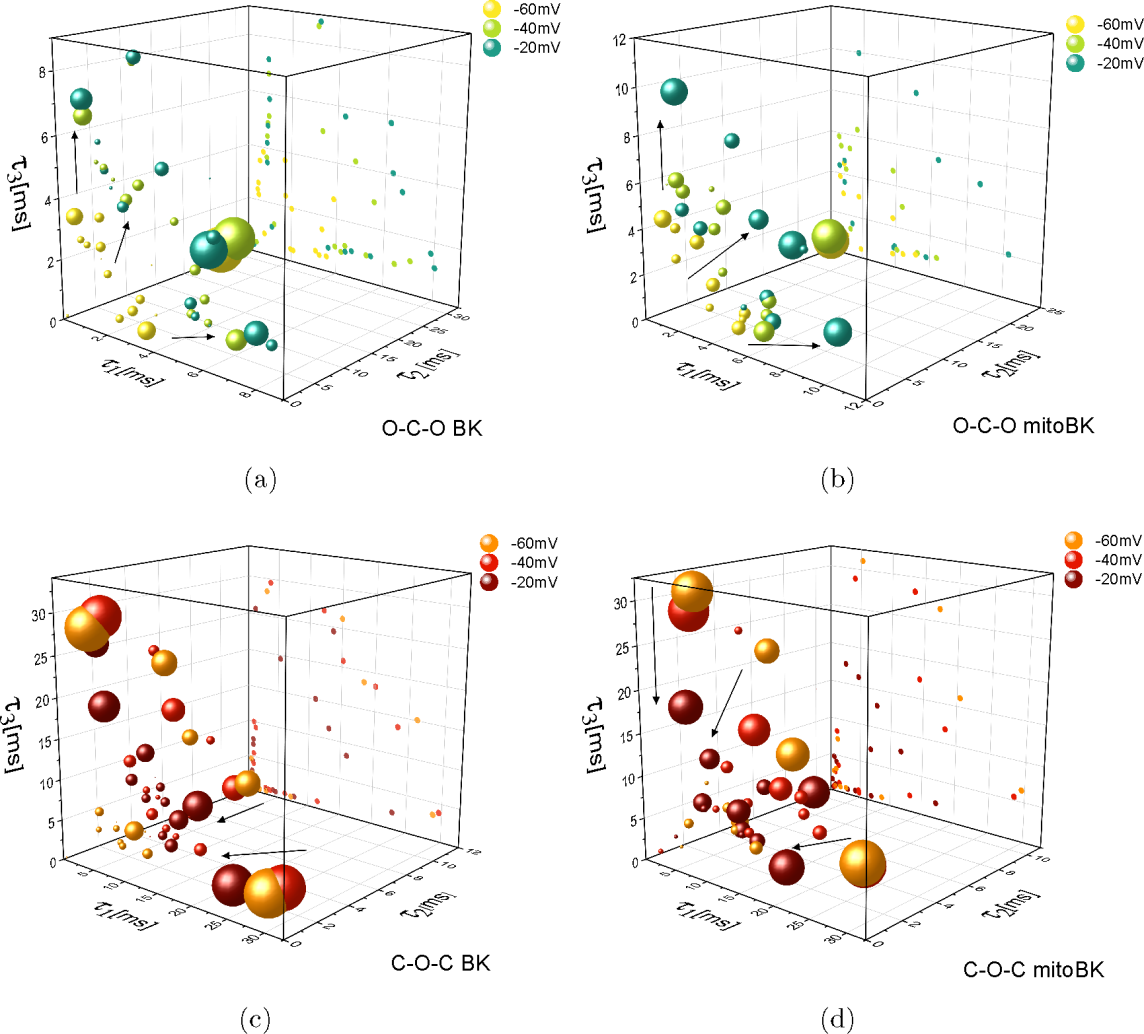
Phase space representation of the clusters of the O–C–O (a, b) and C–O–C (c, d) sequences describing channel gating at membrane hyperpolarization for the cellular (a, c) and mitochondrial (b, d) variants of the BK channels. The results are obtained at *R*_0_ = 0.96 (BK) and *R*_0_ = 0.93 (mitoBK). The coordinates of the centers of the spheres representing the O–C–O sequences are given by the average values of the dwell-times of cross-correlated sequences forming a given cluster. Volumes of the spheres are proportional to the logarithm of its normalized (and rescaled) cardinality. To simplify the recognition of the main directions of the cluster translations with the membrane potential, they are marked with black arrows and the centers of the clusters are orthographically projected on the τ_1_τ_3_ plane and presented by dots in the background.

**Figure 7:**
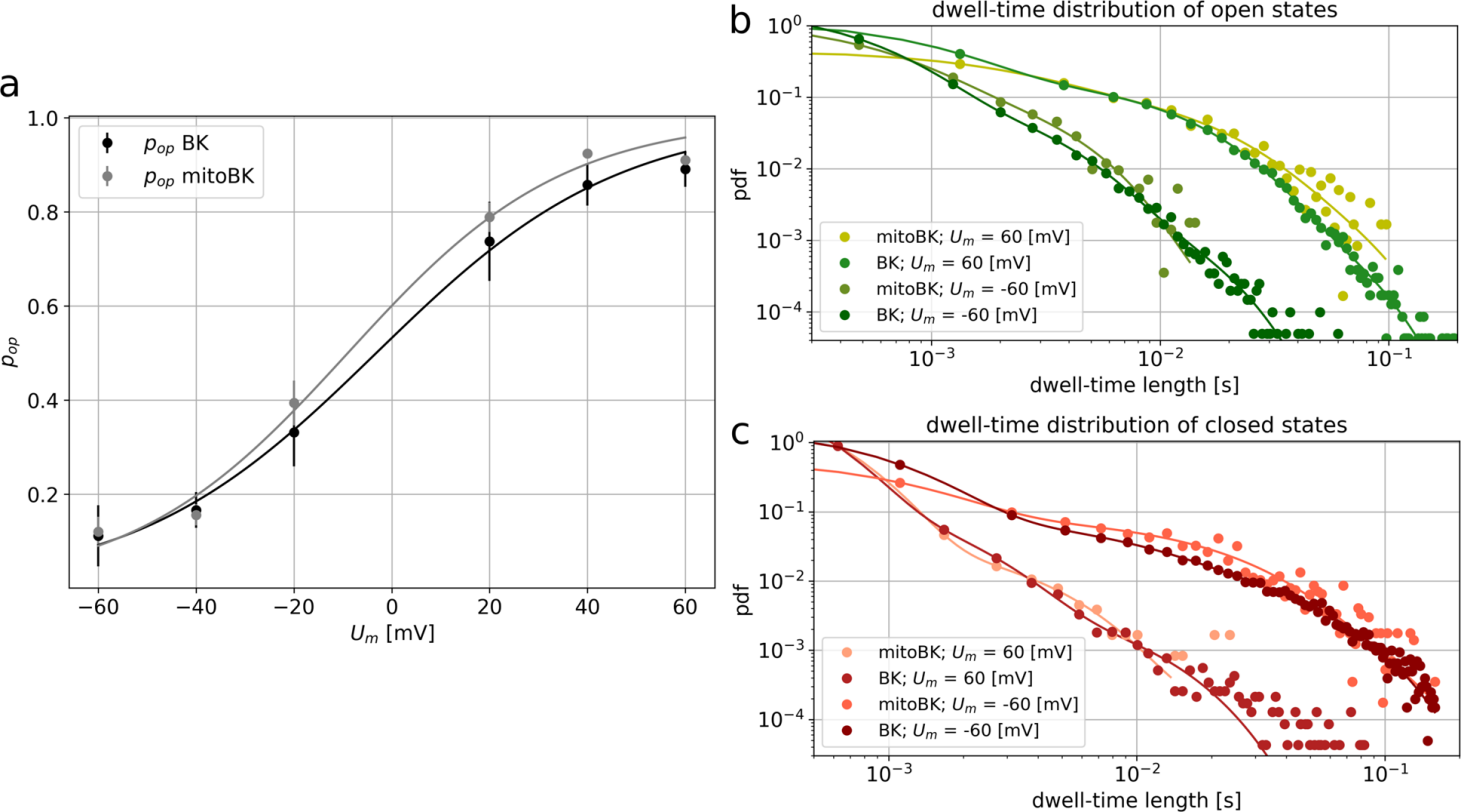
(a) Voltage–activation curves obtained for the BK channel variants from the plasma membrane (BK, black) and the inner mitochondrial membrane (mitoBK, gray). To expose the main tendencies, the experimental data points are shown with appropriate sigmoidal fittings. The error is given as a standard deviation of the mean. *p*_op_ denotes open state probability. *U*_m_ is the membrane potential. The open (b) and closed (c) dwell-time distributions for the mitoBK and BK channels obtained at *U*_m_ = 60 and −60 mV. The appropriate multiexponential fittings are presented together with the corresponding experimental data points.

For the sake of direct visualization, we show here the results obtained for the dwell-time subsequences of *N* = 3 elements. The O–C–O and C–O–C sequences of BK and mitoBK channels at different values of membrane potential are presented in Figures 5 and 6. In the analyzed range of membrane potential, on average, 17 discernible clusters were found for the BK channels and 13 clusters, for the mitoBK channels. The clusters significantly differed by the frequency of its occupation, and ca. 5 dominating ones (reaching above 5% of total occurrences) were recognized for both BK channel variants. Within the results presented in the O–C–O and C–O–C phase space, one can observe “symmetric” tendencies that are imposed by the voltage activation of the channel (Figure 7); i.e., the longer dwell-times of the open states are exhibited at membrane depolarization and relatively long closed dwell-times, at membrane hyperpolarization. In these cases, one can see the well-pronounced “belts” of the frequently occupied O–C–O sequences in Figure 5a,b and C–O–C sequences in Figure 6c,d.

The probability of the occupancy of the dominating clusters of the O–C–O type at membrane depolarization and the C–O–C type at membrane hyperpolarization is more uniformly distributed than in the case of the C–O–C and O–C–O sequences at positive and negative voltages, respectively. At membrane hyperpolarization, one dominant O–C–O sequence can be indicated that reaches over 50% of all occurrences (Figure 6a,b), and an analogous observation can be made for membrane depolarization where one leading C–O–C sequence can easily be detected (Figure 5c,d). In general, the structure of the data corresponding to the C–O–C sequences at high voltages and O–C–O sequences at negative voltages (Figures 5c,d and 6a,b) is more complex in comparison to the structure of the data corresponding to O–C–O sequences at membrane depolarization and C–O–C sequences at membrane hyperpolarization (Figures 5a,b and 6c,d). However, in each case, the main directions of the clusters’ translations with the changes of membrane potential are still visible. As one infers from the results presented in Figures 5 and 6, the overall tendencies within the results obtained for the voltage activation of the BK channels are quite concurrent, which is consistent with the common basic characteristics of the BK-type channels (Figure 7).

The more detailed inspection of the phase space representations of the sets of dwell-time sequences enables us to point out the differences that allow us to discern the BK and mitoBK channel gating. Looking at the structure of the O–C–O sequences at membrane depolarization in Figure 5a,b, one can notice that, within the O–C–O sequences of the *very long–short–short* and *short–short–very long* type, longer open dwell-times are reached for the mitochondrial channel variants (over 20 ms) than for the plasma membrane variants (about 15 ms). In turn, the typical *long–short–long* O–C–O sequences are comparable for the plasma membrane and mitochondrial BK channel variants. The presence of the very long openings in the mitoBK channel gating may find its resemblance in a slight steepening of the mitoBK voltage–activation curve in comparison with the *p*_op_(*U*_m_) curve corresponding to the plasma membrane BK channels (Figure 7a) in a high voltage regime as well as in higher probability density values reached for the long open dwell-times exhibited by the mitoBK in comparison to their plasma membrane counterparts at high voltages (Figure 7b).

For the O–C–O sequences of dwell-times obtained for the plasma membrane BK channels, very short openings are visible in the full range of *U*_m_ although they correspond to the rare events in channel dynamics at membrane depolarization (Figure 5a). The open dwell-times within the O–C–O sequences at membrane hyperpolarization were shorter for the BK channels from the cell membrane than the mitochondrial ones (max of 8 ms vs 12 ms for the BK and mitoBK, respectively), as shown in Figure 6a,b.

In the case of the C–O–C sequences obtained at membrane depolarization in both mitoBK and BK channels, the clusters are accumulated in the range of relatively short closed dwell-times (below 10 ms) (as can be observed in Figure 5c,d), which stems from the voltage activation of the channel (Figure 7a,c). Nevertheless, among the results describing the C–O–C sequences of the BK channels in the cell membrane at positive voltages, one can observe the relatively rare events of long closed sojourns, i.e., lasting up to 30 ms (Figure 5c). The analysis of the structure of the C–O–C sequences at membrane hyperpolarization (in Figure 6c,d) allows one to observe that, although the time scale of the closed dwell-times are comparable (taking values up to 30 ms), the open dwell-times of the mitoBK channels are slightly shorter than the ones corresponding to their cell membrane counterparts (max 10 ms vs 12 ms). The separation of the clusters obtained at −40 and −60 mV is better for the mitoBK channels, which is evident in the τ_1_τ_3_ plane in Figure 6c,d.

## Discussion

In this work, we introduced a relatively simple method of single-channel patch-clamp data examination that is based on cross-correlations within experimental series. Because the proposed methodology focuses on the repetitions of the substate dwell-time sequences of a channel, it directly refers to the connectivity and occupancy of the distinct conformations. The more data available, the more probable it is that we encounter every possible entry/exit path to all stable conformations of the channel.

This method allows us to identify the set of discernible routes between the connected substates within the open and closed state manifolds. In future applications, one can perform such an analysis for the data obtained for different concentrations of channel modulators or drugs. The visual inspection of the effects of drugs on the channel conformational changes in the phase space can enable a closer look than ever before at the behavior of the chemically stimulated channels. It can also be anticipated that monitoring the channels exhibiting pathologies with the appropriate control groups allows one to precisely indicate those sequences of the channel substates that are directly involved in functional channelopathies. Consequently, a search for such drugs that modulate only these pathology-related substates may become desired. Such kind of tactics seems very promising in omitting the side effects of interventional channel activation/blocking exerted by many known channel modulators.

In this work, we focused on the presentation of the main idea of the proposed methodology and the basic principles of its implementation, skipping many important side topics that would darken the presented picture. For clarity and to find the most evident representation of the results, we decided on the 3-dimensional description of the channel activity. The proper thorough inspection of the cluster’s structure and dispersion and their mutual dependencies as well as providing further channel gating descriptions for higher dimensionality (*N* > 3) and connectivity analysis between correlations bring new broad fields of exploitation of the introduced methodology.

Our research underscores the informative character of the analysis of dwell-time sequences due to their correspondence to the unique entry/exit routes to the distinct conformations of the channel. The investigations of the stimulus-related changes of the diagram representing the channel’s dynamics in a dual space is interesting on its own. Nevertheless, it also brings a new perspective to further develop such approaches and utilize them in the reconstruction of the Markovian model that can describe the channel dynamics. The analysis of the correlations and repetitiveness of the dwell-time sequences can be exploited in the search of the number of and possible connections between the available substates of the channel gating. To some extent, it was already postulated by the pioneers in the field who tried to estimate the number of entry routes into the functional states by investigating the correlations between successive dwell-times.^2,3,6^ Nevertheless, to achieve successful performance of the kinetic model reconstruction, one should probably work out advanced procedures for the separation and unique identification of those dwell-times for which their exponential dwell-time distributions overlap. In this work, such problematic dwell-times were simply handled by the recognition of the correlated dwell-time sequences starting from the tails of the distributions. Hence, we inspected the descendingly sorted lists of dwell-time subseries for the presence of sufficiently high cross-correlation. It is enough to group the similar, repetitive, and cross-correlated dwell-time sequences and observe the main tendencies (Figures 5 and 6). Nevertheless, other, more sophisticated approaches to the dwell-time classification should be used in kinetic model reconstruction. They could involve, for example, transformations of the dwell-times to random variables displaying more tractable probability distributions (like a known transformation from the exponential distribution to a nonzero maximum displaying Weibull distribution) or extraction of additional physical features of the current signal, which discriminate between similar dwell-times generated in different states (where the interaction with a different surrounding must leave some fingerprint on the current traces).

As a practical application of the introduced method, we compared the characteristics of the BK and mitoBK channel activity for which a very close voltage–activation level was reached (Figure 7). It turned out that the number of clusters and their location were different for the two analyzed channel variants. There are two possible factors that can be used to explain these differences. First, there are structural and functional differences between the BK and mitoBK channels. Although both channel variants are encoded by the same gene (Kcnma1), the mitochondrial variants of the channel are expressed when this gene undergoes splicing to the DEC isoform characterized by the additional 50-aa C-terminal sequence.^20^ According to the literature, the BK isoform found in the cell membrane and the BK-DEC splice variant exhibit functional changes, which are represented by some discrepancies in the gating characteristics.^4,11^ For example, the mitoBK channels exhibit a higher sensitivity to Ca^2+^ ions, which forced us to use different calcium concentrations in the appropriate patch-clamp experiments to reach comparable voltage–activation curves; please see Figure 7. Additionally, besides the structural changes between the BK and mitoBK channel variants, also the differences in biophysical and biochemical properties between plasma and mitochondrial membrane can contribute to the recognized differences in channel gating.^21−25^ The cell membrane is highly saturated and contains relatively large amounts of sphingomyelin and cholesterol in contrast to the inner mitochondrial membrane, which is characterized by a high degree of unsaturation, the presence of cardiolipin, and the absence of cholesterol.^26^

Second, due to technical difficulties, the data sets describing the BK channels from the plasma membrane and mitochondrial patches were not equal in size. Due to the fact that the total length of the dwell-time series of the plasma membrane BK channels was over 4 times longer than the one corresponding to the mitochondrial channels, for the mitoBK channels, one has a smaller chance to observe relatively rare states (e.g., short-lasting open states at membrane depolarization). Thus, a different total number of clusters was observed for the mitoBK and BK channels. Still, however, these differences refer to the sparsely occupied sequences (below 5% of all occurrences).

## Conclusions

In this work, we have presented a new method for the analysis of connectivity of distinct channel substates. According to this approach, the cross-correlations within the dwell-time series turn out to be useful to unravel the ion channels’ conformational dynamics and allow the effective representation of channel gating by a diagram in a phase space. We are convinced that the proposed methodology creates a new field of exploration for the research on the ion channel’s activity, since the phase space diagrams are informative yet easier to analyze than raw patch-clamp data, especially when the modern computational techniques of cluster analysis would be implemented.

## Notes

The software and metadata are available at https://zenodo.org/record/6340407#.YpaT46jMJPY.
